# Isolation and Enhancement of a Homogenous *in Vitro* Human Hertwig’s Epithelial Root Sheath Cell Population

**DOI:** 10.3390/ijms140611157

**Published:** 2013-05-27

**Authors:** Manal Farea, Ahmad Sukari Halim, Nurul Asma Abdullah, Chin Keong Lim, Khairani Idah Mokhtar, Zurairah Berahim, Kasmawati Mokhtar, Abdul Qawee Rani, Adam Husein

**Affiliations:** 1 Conservative Department, School of Dental Sciences, Universiti Sains Malaysia, 16150 Kubang Kerian, Kelantan, Malaysia; E-Mails: zurairah@kb.usm.my (Z.B.); hidayah@kb.usm.my (K.M.); 2 Reconstructive Sciences Unit, School of Medical Sciences, Universiti Sains Malaysia, 16150 Kubang Kerian, Kelantan, Malaysia; 3 Oral Biology Unit, School of Dental Sciences, Universiti Sains Malaysia, 16150 Kubang Kerian, Kelantan, Malaysia; E-Mails: nurulasma@kk.usm.my (N.A.A.); khairani@kk.usm.my (K.I.M.); 4 Centre of Studies for Preclinical Science, Faculty of Dentistry, Universiti Teknologi MARA, 40450 Shah Alam, Selangor, Malaysia; E-Mail: lim125@hotmail.com; 5 Human Genome Centre, School of Medical Sciences, Universiti Sains Malaysia, 16150 Kubang Kerian, Kelantan, Malaysia; E-Mail: rcrc44@yahoo.com

**Keywords:** Hertwig’s epithelial root sheath (HERS), homogenous cell culture, selective digestion, characterization

## Abstract

Hertwig’s epithelial root sheath (HERS) cells play a pivotal role during root formation of the tooth and are able to form cementum-like tissue. The aim of the present study was to establish a HERS cell line for molecular and biochemical studies using a selective digestion method. Selective digestion was performed by the application of trypsin-EDTA for 2 min, which led to the detachment of fibroblast-like-cells, with the rounded cells attached to the culture plate. The HERS cells displayed a typical cuboidal/squamous-shaped appearance. Characterization of the HERS cells using immunofluorescence staining and flow cytometry analysis showed that these cells expressed *pan-cytokeratin*, *E-cadherin*, and *p63* as epithelial markers. Moreover, RT-PCR confirmed that these cells expressed epithelial-related genes, such as *cytokeratin 14*, *E-cadherin*, and *ΔNp63*. Additionally, HERS cells showed low expression of CD44 and CD105 with absence of CD34 and amelogenin expressions. In conclusion, HERS cells have been successfully isolated using a selective digestion method, thus enabling future studies on the roles of these cells in the formation of cementum-like tissue *in vitro*.

## 1. Introduction

Tooth development and periodontal tissue formation are regulated by a series of reciprocal interactions between the oral epithelium and mesenchymal cells. During tooth development, after crown formation, the apical mesenchyme continues to proliferate to form the developing periodontium while the inner and outer enamel epithelial cells fuse below the level of the cervical margin of the crown to develop a bi-layered epithelial sheath, which is called Hertwig’s epithelial root sheath (HERS) [1]. After root formation, the epithelial sheath fenestrates and individual cells migrate away from the root into the region of the future periodontal ligament to form the epithelial rests of Malassez (ERM) or undergo apoptosis [2]. HERS cells are a unique population of epithelial cells in the periodontal ligament that play a pivotal role in cementum repair [3], and it has been reported that HERS cells can differentiate into cementoblast-like cells through the epithelial-mesenchymal transition (EMT) [4].

Heterogeneous culture may cause variations in results due to differential expression in different cells. In a study by Sonoyama *et al*. (2007) [4], the periodontal ligament (PDL) tissue was digested with collagenase and dispase for the isolation of HERS cells prior to the single-cell suspensions being seeded and grown in a defined keratinocyte- serum-free medium (SFM). However, these authors reported that the HERS cells were sub-cultured only once for use in their experiments, a procedure that might not yield a homogenous population and might have compromised the accuracy of the interpretation of the results. In fact, the primary culture exhibited different cell populations, with a fibroblast-like appearance and rounded/squamous epithelial cell-like appearance [5–7], indicating that the primary culture did not contain pure HERS cells because fibroblast-like cells will not be removed from merely one passage. Therefore, a primary culture needs to be either sub-cultured several times until the fibroblast-like cells are eliminated [5–8] or processed with the use of a magnetic-activated cell sorting system to obtain a homogenous cell culture from the mixed cell population [9].

Although several *in vitro* studies have been performed to isolate and characterize HERS cells and certain derivative cell lines [4,7,8,10,11], none has attempted to establish a homogenous HERS cells in culture. Therefore, the aim of the present study was to isolate and culture primary HERS cells using a newly developed selective digestion method to develop a homogenous HERS cell line.

## 2. Results

### 2.1. Primary Isolation of HERS Cells

**Figure 1:**
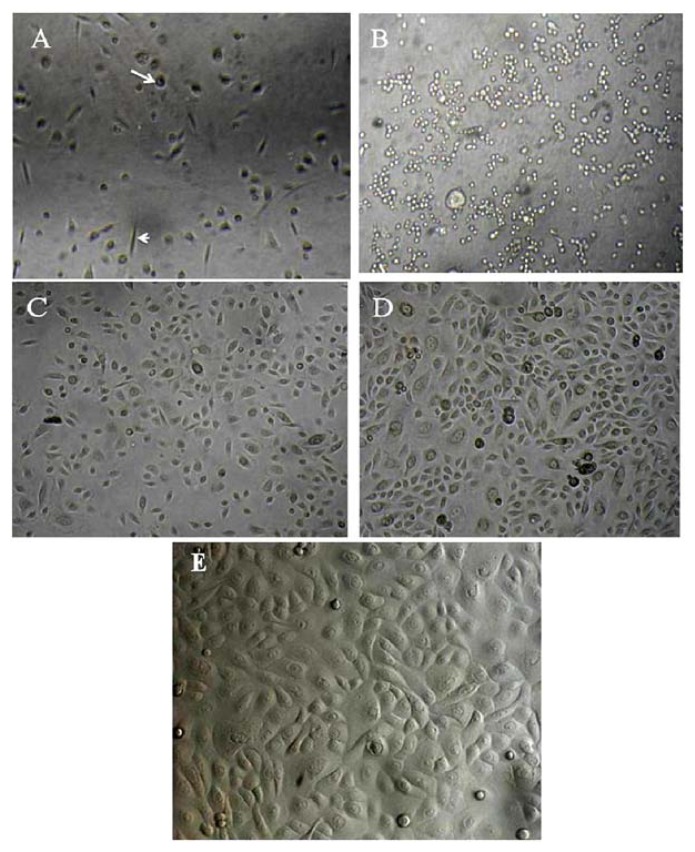
Primary culture (P0): heterogeneous cells, rounded cells (arrow), and fibroblast-like cells (arrowheads) (**A**); Attached epithelial-like cells after the selective digestion method (**B**); One day after the selective digestion (**C**); Colony formation began at day 7 (**D**); and the cells became confluent by day 10 (**E**). Magnification is 20×.

Periodontal ligaments were digested in a mixture of collagenase and dispase, and the single-cell suspensions were seeded in KGM. The primary cultures consisted of cells with a fibroblast-like appearance and rounded/squamous epithelial cell-like appearance (Figure 1A). Therefore the primary heterogeneous cells at first passage were further processed using a selective digestion method (application of trypsin-EDTA for 2 min). This treatment resulted in the detachment of the fibroblast-like cells from the surface of the culture plates and is based on the observation that epithelial-like cells are always attached more strongly than fibroblast-like cells (Figure 1B). The attached epithelial-like cells were then further cultured in KGM, yielding a more homogenous culture consisting mostly of cobblestone/squamous-shaped epithelial cells (Figure 1C). The number of cells increased at day 7 following the selective digestion, and reached confluence within 10–14 days, with most cells showing a typical epithelial-like shape (Figure 1D,E). Interestingly, the cells continued to proliferate and grow well until the sixth passage, but ceased to proliferate thereafter.

### 2.2. Immunofluorescence Staining of HERS Cells

**Figure 2:**
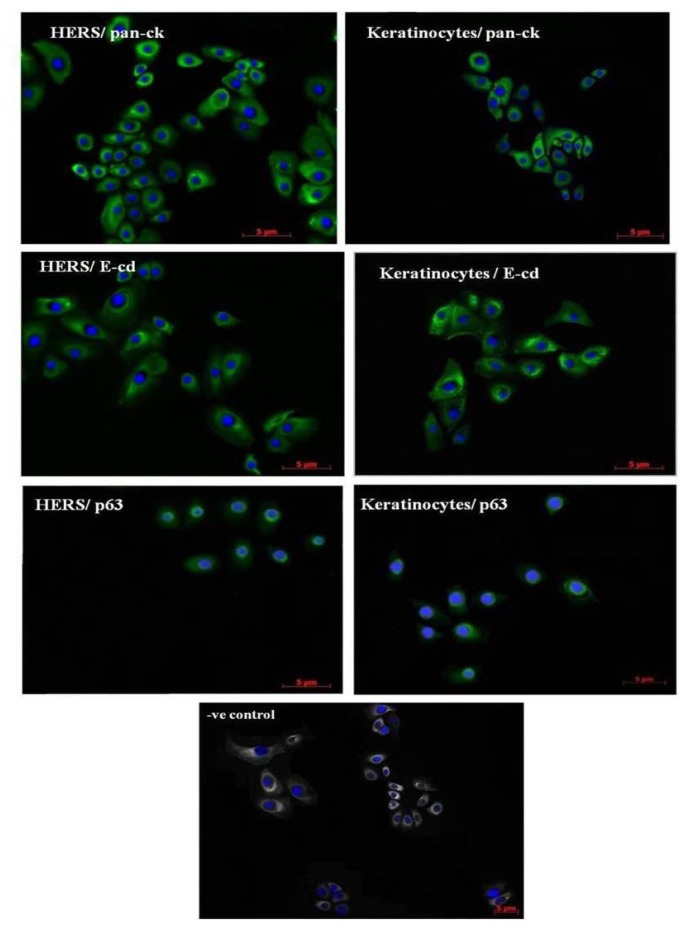
Immunofluorescence staining of HERS cells and keratinocytes for pan-cytokeratin (pan-ck), E-cadherin (E-cd), and p63. The nuclei were counterstained with DAPI. The negative control showed no signal, which supports the validity of our staining. Scale bars = 5 μm, magnification is 40×.

Immunofluorescence staining was conducted to characterize the HERS cells *in vitro*. The culture was positively stained using antibodies against pan-cytokeratin, E-cadherin, and p63. The same expression pattern was found in keratinocytes (Figure 2).

### 2.3. Flow Cytometry Analysis of HERS Cells

**Figure 3:**
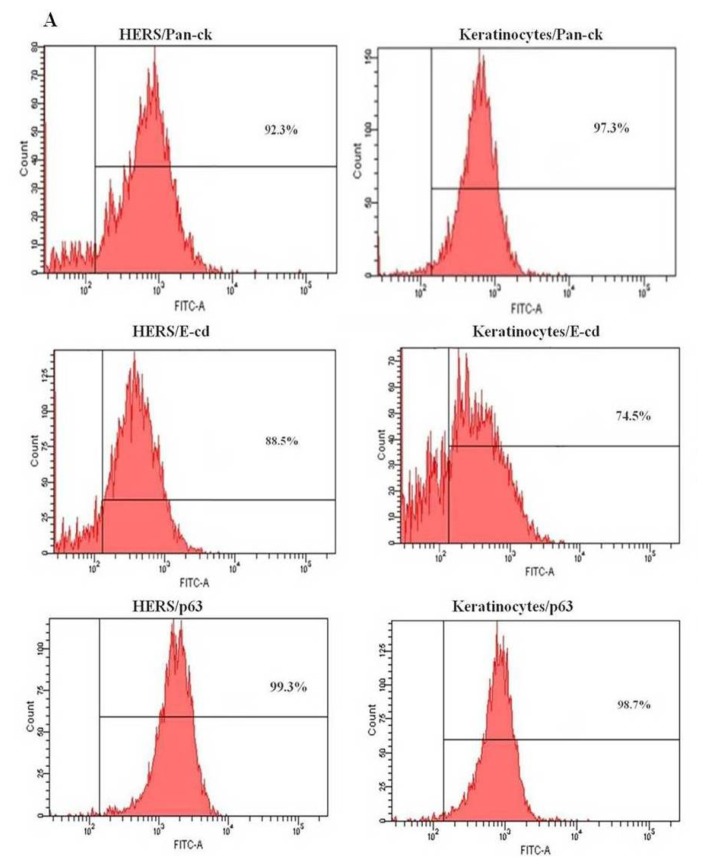

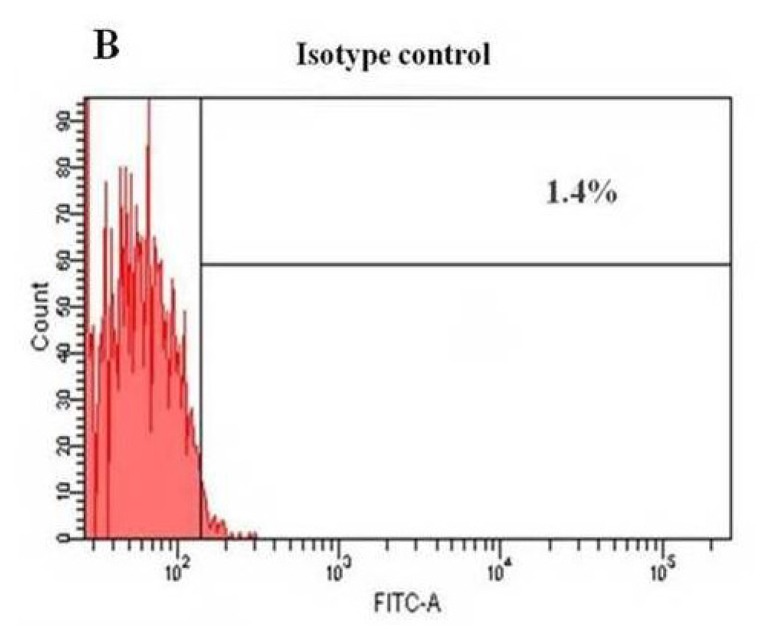
Expression profiles of HERS cells and keratinocytes by flow cytometry analysis. The two cell populations display similar expression for pan-cytokeratin (pan-ck), E-cadherin (E-cd), and p63 (**A**); Isotype control (IgG1) (**B**).

**Figure 4:**
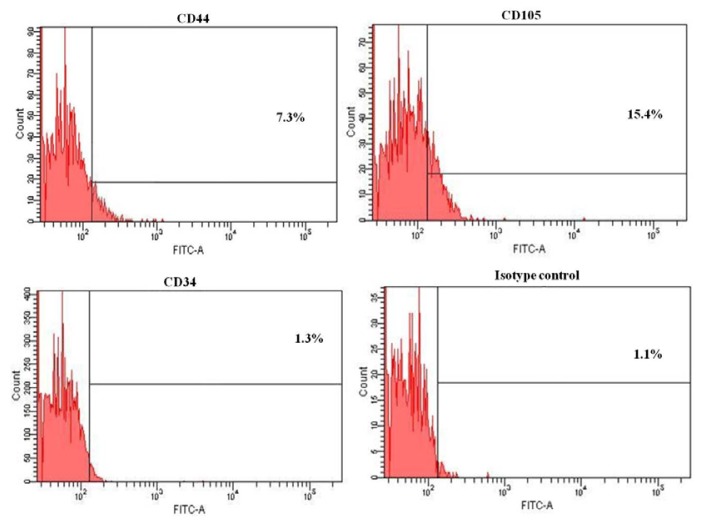
Expression profile of HERS cells by flow cytometry analysis. HERS cells display low expression of mesenchymal markers (CD44 and CD105) with absence of CD34 marker.

To further characterize the HERS cells, the expression pattern of the three epithelial markers, *pan-cytokeratin*, *E-cadherin*, and *p63*, was examined using flow cytometry at passage 3. E-cadherin and p63 were more abundant in the HERS cells than keratinocytes (Figure 3A,B). However, E-cadherin was less expressed in both HERS cells (88.5%) and keratinocytes (74.5%) compared to pan-cytokeratin (92.3% and 97.3%, respectively) and p63 (99.3% and 98.7%, respectively). Moreover, HERS cells showed low expression of mesenchymal markers such as CD44 (7.3%) and CD105 (15.4%). However, the endothelial marker CD34 (1.3%) was not detected (Figure 4). This proves that HERS cells were neither mesenchymal nor endothelial cells.

### 2.4. sqRT-PCR

**Figure 5:**
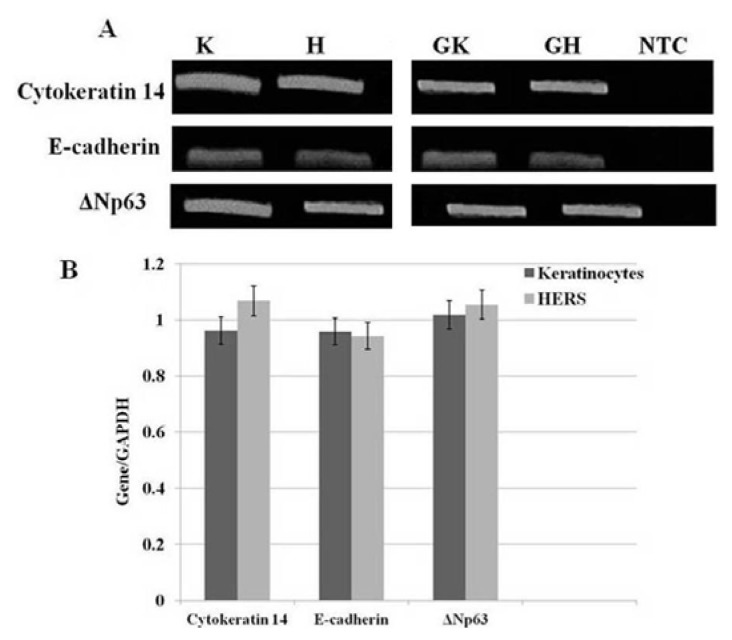
Gene expression of *cytokeratin 14*, *E-cadherin*, and *ΔNp63* in HERS cells (H) and keratinocytes (K) (**A**). Both cell types were positive for the presence of *cytokeratin*, *E-cadherin*, and *ΔNp63. GAPDH* in keratinocytes (GK), *GAPDH* in HERS (GH). No-template control (NTC) consisting of distilled water in place of template. Semi-quantitative RT-PCR analysis of the *cytokeratin 14*, *E-cadherin*, and *ΔNp63* genes in HERS cells and keratinocytes (**B**). The expression level of each gene was normalized to the corresponding GAPDH endogenous control. The data showed no significant differences between the groups.

**Figure 6:**
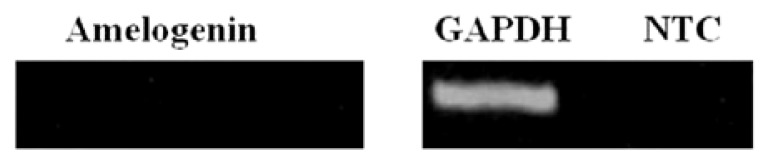
Absence of amelogenin expression in HERS cells. The expression level of amelogenin gene was normalized to the corresponding *GAPDH* endogenous control.

sqRT-PCR was performed to examine the gene expression of *cytokeratin 14*, *E-cadherin*, and *ΔNp63* in both HERS cells and keratinocytes. The RT-PCR analysis showed that these three genes were highly expressed in both types of cells (Figure 5). Moreover, the expression of amelogenin in HERS cells was not detected (Figure 6).

## 3. Discussion

The human periodontium serves as a supporting structure for teeth and consists of the periodontal ligament, mucosa, alveolar bone, cementum, and a wide variety of cells. HERS cells are unique epithelial cells compared with the other components of the dental epithelium. HERS cells remain in the periodontal tissues throughout the life of the adult and help to maintain the homeostasis of the periodontium by reciprocal interactions with other periodontal cells, prevent root resorption and induce acellular cementum formation [12]. Moreover, it is reported that HERS cells undergo EMT and produce cementum-forming cells [4,13]. In addition, HERS cells control the differentiation of periodontal ligament stem cells (PDLSc) [4].

The epithelial markers that have been proposed to identify epithelial cells can be categorized into three groups: nuclear proteins, such as p63; cell membrane proteins, such as E-cadherin; and cytoplasmic proteins, such as pan-cytokeratin [4,5,11,14]. Both keratin 14 and p63 are highly active in immortalized oral keratinocyte cells (IMOK cells) [15], and several studies have reported that keratin, p63, and E-cadherin are highly expressed in human epidermal keratinocytes [16–19]. Additionally, both oral and epidermal keratinocytes express these epithelial markers [20,21]. Therefore, human epidermal keratinocytes were used as a positive control in this study.

This study is the first report of the successful establishment of HERS cells using a new technique, selective digestion, to obtain pure HERS cells population from a primary heterogeneous cell culture after several attempts and optimizations. Based on the observation that epithelial cells attach more strongly to culture plates than fibroblast-like cells, the selective digestion method employs 0.25% trypsin-EDTA for 2 min. However, the prolonged exposure to trypsin can affect the integrity of the cell membrane, cell viability, and ultimately lead to cell necrosis [22,23]. Indeed, Sutradhar *et al*. (2010) [24] found that trypsinization for 5 min with 0.25% trypsin-EDTA did not have any significant detrimental effect on the chondrocyte membrane or cell viability, whereas 20 min of trypsinization had a potent deleterious effect on both the cell membrane and viability.

The selective digestion method was very efficient in promoting the detachment of the fibroblast-like cells in the initial heterogeneous culture, leaving the epithelial-like cells adhered to the culture plate. Thereafter, the HERS cells were cultured in KGM, which is specifically used to culture epithelial cells; hence, the few remaining fibroblast-like cells were removed from the culture by the subsequent sub-culturing processes. This result proves that KGM was specific to grow only epithelial cells [5,8]. Non-epithelial cells may be transiently viable but won’t proliferate in the culture and they are diminished in the subsequent culture. Furthermore, KGM medium was the first medium reported to cultivate the cells [8]. Although Sonoyama *et al*. (2007) [4] reported that a keratinocyte-serum-free medium (K-SFM) was used to avoid PDLSC contamination of HERS cells, in our study, K-SFM was tried and found that it allowed the active proliferation of fibroblast-like cells.

Under our conditions, the morphology of the HERS cells was that of typical cuboidal/cobblestone-shaped epithelial cells, which is in agreement with other studies [4,8,10,11]. Akimoto *et al*. (2011) [11] reported that their immortalized HERS cell line presented a cobblestone-like appearance and retained their proliferation activity up to 60 passages. However, we found that the HERS cells sustained their proliferation activity only until sixth passage, which corroborates a study by Nam and Lee (2009) [5]. In contrast, Sonoyama *et al*. (2007) [4] stated that their HERS cell line was applied at first passage in their experiments, a condition in which we consistently noted that the cells were not comprised of pure epithelial-like cells and required further sub-culturing. Indeed, a homogenous culture is crucial in an experiment, as a heterogeneous culture may cause variation in the results due to genes that are differentially expressed by different cells [25–27].

Our immunofluorescence results showed that the HERS cells expressed the tested epithelial markers, whereas no staining was identified in the negative control, confirming the validity of our protocol, and that the staining was not caused by non-specific interactions of immunoglobulin molecules with the cells. Moreover, flow cytometry and RT-PCR results confirmed that the HERS cells positively expressed pan-cytokeratin, p63, and E-cadherin as epithelial markers and could be characterized as epithelial-like cells, consistent with other studies [5,7,11]. Interestingly, p63 was found to be highly expressed in both the HERS cells and keratinocytes according to the flow cytometry and RT-PCR analyses. Several studies have reported that the p63 protein, and its truncated dominant-negative isoform, ΔNp63, are present in the basal cells of many human epithelial tissues [16,17,28–30]. Moreover, high expression levels of the p63 protein have also been reported in actively proliferating keratinocytes [16,17]. In the flow cytometry analysis, HERS cells displayed low expression of CD44 and CD105 as mesenchymal markers with no detectable expression of CD34 as endothelial marker. This confirms that HERS cells were neither mesenchymal nor endothelial cells, but they were epithelial-like cells, corroborating a study by Nam *et al.* (2011) [7]. The possibility of contamination with other epithelial cells as ameloblasts or preameloblasts was ruled out by the absence of amelogenin (a major protein of enamel that is secreted by ameloblasts [31–33]) expression, which further supports previous *in vitro* [8] and *in vivo* [34,35] studies. Taken together, our findings support the purity of our established HERS cells which showed the typical appearance of epithelial cells and positively expressed pan-cytokeratin, E-cadherin, and p63 as epithelial markers.

HERS cells are a unique epithelial population that plays a critical role during root formation, thus the establishment of a cell line is essential and would serve as an *in vitro* model for future studies.

## 4. Experimental Section

### 4.1. Primary Isolation and Culture of Human HERS Cells

**Figure 7:**
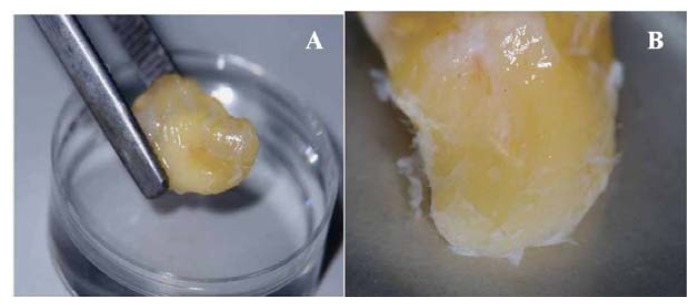

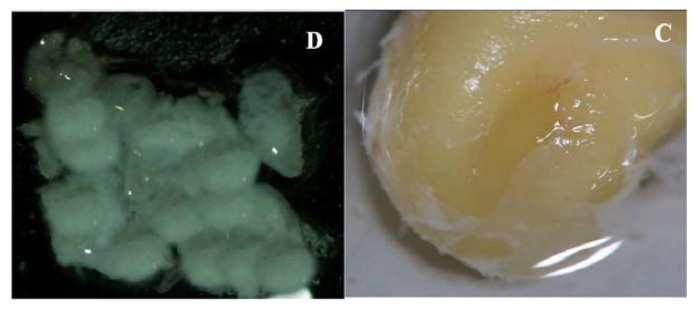
Microscopic view of the extracted human impacted third molar (**A**), periodontal ligament (PDL) attached to the root surfaces (**B**,**C**), and the isolated and minced tissues (**D**).

Normal, freshly extracted human impacted third molars (*n* = 40) were collected from adults, aged between 18 and 23 years old, who attended dental clinics of the School of Dental Sciences, Universiti Sains Malaysia, USM, Malaysia. The study was approved by the Human Research Ethics Committee of Universiti Sains Malaysia, USM, Malaysia. The teeth were delivered in Hank’s balanced salt solution (Gibco, Carlsbad, CA, USA) supplemented with 3% antibiotics/antimycotics (Gibco, Carlsbad, CA, USA). The periodontal ligament (PDL) tissues were gently separated from the root surfaces of the teeth using a fine forceps (Figure 7). The isolated tissues were minced and digested in a solution of 3 mg/mL collagenase type I (Gibco, Carlsbad, CA, USA) and 4 mg/mL dispase (Gibco, Carlsbad, CA, USA) for 1 h at 37 °C to release the cells into a single-cell suspension. The cells were seeded in 6-well culture plates with 3 mL of serum-free keratinocyte growth medium (KGM) (Lonza, Walkersville, MD, USA). The medium was changed every 48 h after the initial plating. The primary heterogeneous cell culture was further processed using a selective digestion method with the application of 0.25% trypsin-EDTA (Gibco, Carlsbad, CA, USA) for 2 min, yielding a detachment of the fibroblast-like-cells, and leaving the rounded-like cells attached to the culture plate. This latter population was further cultured in KGM and upon reaching 70% confluence, the cells were sub-cultured, counted, and photographed.

### 4.2. Indirect Immunofluorescence Staining

HERS cells (at passage 3) were cultured in 4-well culture slides until they reached 70% confluence. After washing with phosphate-buffered saline (PBS) three times for 5 min each, the cells were then fixed with freshly prepared 4% paraformaldehyde (PAF) diluted with PBS for 10 min at room temperature (RT). The fixative solution was aspirated, and the cells were washed with PBS before being blocked with 10% normal goat serum for 1 h at RT. The following antibodies were used: monoclonal mouse anti-human Pan-cytokeratin (Abcam^®^, Cambridge, UK) (1:300), anti-E-cadherin (Abcam^®^, Cambridge, UK) (1:50), and anti-p63 (Santa Cruz Biotechnology Inc., CA, USA) (1:100). For the antibodies against intracellular and nuclear proteins, a permeabilization solution (0.1% Triton X-100/PBS) was applied to the cells for 5 min. The primary antibodies were diluted in PBS + 10% goat serum and applied to the cells overnight at 4 °C. After washing with PBS three times for 5 min each, goat anti-mouse fluorescein (FITC)-conjugated secondary antibody 1 gG (1:200) (Abcam^®^, Cambridge, UK) was applied, and the samples were incubated in the dark for 1 h at RT. Following this incubation, the cells were washed three times with PBS containing 0.1% Tween-20 and counterstained with 4′-6-diamidino-2-phenylindole (DAPI) (Sigma-Aldrich, St. Louis, MO, USA) for 15 min; the cells were then washed with PBS. The slides were mounted in a fluorescent mounting medium (DAKO) before being viewed under a fluorescence image analyzer (Zeiss, Oberkochen, Germeny). The experiment was performed in triplicate. Human epidermal keratinocytes (obtained from the Reconstructive Unit, School of Medical Sciences, Universiti Sains Malaysia) were used as a positive control and isotype mouse IgG1as a negative control.

### 4.3. Flow Cytometry Analysis

For the flow cytometry analysis, the HERS cells were harvested at passage 3 and washed with PBS. The following antibodies were used: monoclonal mouse anti-human Pan-cytokeratin (Abcam^®^, Cambridge, UK) (0.2 μL); anti-E-cadherin (Abcam^®^, Cambridge, UK) (20 μL); and anti-p63 (Santa Cruz Biotechnology Inc., CA, USA) (1 μL). Each primary antibody was incubated with 1 × 10^6^ cells for 1 h in the dark at RT. After washing with PBS, goat anti-mouse fluorescein (FITC)-conjugated secondary antibody IgG (1:100) was applied for 30 min in the dark at RT. The cells were then resuspended in PBS solution and analyzed using flow cytometry (FACSCanto II, Becton, Dickinson and Company, Franklin Lakes, NJ, USA). For the antibodies against intracellular and nuclear proteins, the cells were fixed with 1% paraformaldehyde for 10 min and permeabilized with ice-cold methanol for 10 min before incubation with the primary antibody. Keratinocytes were used as a positive control and isotype mouse IgG1 as a negative control. To further characterize HERS cells, mesenchymal markers were utilized following the same protocol: monoclonal mouse anti-human CD44 (Abcam^®^, Cambridge, UK) (1 μL); anti CD105 (Abcam^®^, Cambridge, UK) (10 μL); endothelial marker monoclonal mouse anti-human CD34 (Abcam^®^, Cambridge, UK) (10 μL).

### 4.4. Semi-Quantitative Reverse Transcription-Polymerase Chain Reaction (sqRT-PCR)

The HERS and keratinocytes were harvested at passage 3, and the total RNA was extracted using the RNeasy kit (Qiagen, Valencia, CA, USA). Then, 1 μg of total RNA was reverse transcribed using SuperScript^®^ VILO™ cDNA Synthesis Kit (Invitrogen, Carlsbad, CA, USA) according to the manufacturer’s instructions. The obtained cDNA was used as the template for PCR. A housekeeping gene (*GAPDH*) was used to normalize all the reactions, and a no-template control (NTC) was included in all the reactions. Human-specific primers for the *cytokeratin 14*, *E-cadherin*, *amelogenin*, and *GAPDH* genes were designed according to the published cDNA sequences in GenBank, and the sequence for *ΔNp63* as reported by Yang *et al*. 1998 [30] (Table 1). The cycling conditions consisted of an initial denaturation at 95 °C for 5 min, followed by 30 cycles of denaturation at 95 °C for 1 min, primer annealing at 60 °C for 1 min for *cytokeratin 14* and *E-cadherin* and 61.5 °C for *p63*, and 1 min of extension at 72 °C, followed by 1 step of a final extension at 72 °C for 5 min. The PCR products were separated on a 2% agarose gel and visualized using SYBR Green. The band intensities were then quantified using alpha digital software. The results were normalized to *GAPDH*, and compared as a ratio of each expressed gene. All the reactions were performed in triplicate.

## 5. Conclusions

In conclusion, selective digestion was proven to be a useful method to produce a homogenous culture of HERS cells. Moreover, the HERS cells were positive for epithelial markers (pan-cytokeratin, E-cadherin, and p63). Thus, our data showed that HERS cells were successfully established, and their purity and identity were confirmed both morphologically and biochemically.

## Figures and Tables

**Table 1:** Sequence of primers used for RT-PCR.

Gene	Sequence
*Cytokeratin14*	5′-GTCCTTCTGCAGATTGACAATGA-3′ (forward) 5′-TTCACCAAGACAGAGGAGCTGAA-3′ (reverse)
*E-cadherin*	5′-GAAGATTGCACCGGTCGACAAA-3′ (forward) 5′-CCACCAAAGTCACGCTGAATAC-3′ (reverse)
*ΔNp63*	5′-CAGACTCAATTTAGTGAG-3′ (forward) 5′-AGCTCATGGTTGGGGCAC-3′ (reverse)
*Amelogenin*	5′-GGAGCAGCTTTTGCCATGCCT-3′ (forward) 5′-GTGGCCTTATGCTCTGGTAC-3′ (reverse)
*GAPDH*	5′-GAGTCAACGGATTTGGTCGT-3′ (forward) 5′-GACAAGCTTCCCGTTCTCAG-3′ (reverse)
